# Intelligent Monitoring Model for Fall Risks of Hospitalized Elderly Patients

**DOI:** 10.3390/healthcare10101896

**Published:** 2022-09-28

**Authors:** Amal H. Alharbi, Hanan A. Hosni Mahmoud

**Affiliations:** Department of Computer Sciences, College of Computer and Information Sciences, Princess Nourah Bint Abdulrahman University, P.O. Box 84428, Riyadh 11671, Saudi Arabia

**Keywords:** deep learning, elderly patients, fall risks, classification

## Abstract

Early detection of high fall risk is an important process of fall prevention in hospitalized elderly patients. Hospitalized elderly patients can face several falling risks. Monitoring systems can be utilized to protect health and lives, and monitoring models can be less effective if the alarm is not invoked in real time. Therefore, in this paper we propose a monitoring prediction system that incorporates artificial intelligence. The proposed system utilizes a scalable clustering technique, namely the Catboost method, for binary classification. These techniques are executed on the Snowflake platform to rapidly predict safe and risky incidence for hospitalized elderly patients. A later stage employs a deep learning model (DNN) that is based on a convolutional neural network (CNN). Risky incidences are further classified into various monitoring alert types (falls, falls with broken bones, falls that lead to death). At this phase, the model employs adaptive sampling techniques to elucidate the unbalanced overfitting in the datasets. A performance study utilizes the benchmarks datasets, namely SERV-112 and SV-S2017 of the image sequences for assessing accuracy. The simulation depicts that the system has higher true positive counts in case of all health-related risk incidences. The proposed system depicts real-time classification speed with lower training time. The performance of the proposed multi-risk prediction is high at 87.4% in the SERV-112 dataset and 98.71% in the SV-S2017 dataset.

## 1. Introduction

Falls are indicators of feebleness, immovability, and severe chronic injury in elderly people. Fall risks reduce normal tasks by producing injury and movement limitations. Most injuries in the elderly are caused by falls that can produce hip fractures and forearm injuries [1,2,3].

The prediction of fall risks must consider the range of ages and fitness status within the elderly patients and report the variety of reasons for falls. Fall risk factors are listed in controlled studies, which agree to the detection of patients at risk. Older people with several health deficiencies are at the highest risk [4,5,6].

A fall is defined as an unintentional occasion that leads the patient to rest on a ground level. Falls are designated in three stages, as follows [7]:

- An introducing event that relocates the patient’s center of mass away from its base of support which can be caused by environmental hazards or unstable joint weakness.
- The second stage of a fall is the inability of the patient to sustain an upright posture to perceive this movement in time to evade a fall due to loss of sensory function.
- The third stage is the effect of the patient’s body on solid surfaces, which yields to the conduction of forces to the patients’ organs. The prospective for damage is a function of the magnitude and direction of the fall forces.

Research is moving towards automation, deep learning (DL), and artificial intelligence. Dynamic monitoring networks speed up the stream of health-related risk information [1,2,3]. These developments convey convenience to high-risk situations and reveal concealed dangers to hospitalized elderly patients. Thus, protecting people’s lives from violation or violence incidents is a life-risking challenge and is a needed research track for monitoring research. There are numerous health-related risks to personal lives and hospitals everywhere. In [2], the authors studied three metallic materials, and their applications in metal-on-metal bearing for hip implants in terms of contact pressure. The authors employed finite element simulation for predicting contact pressure under normal walking conditions. Recently, both the health-related risk techniques and the count of monitoring health-related risks have grown radically. This carries random risks to the safety and stable process of many premises such as hospitals and hospices. Thus, effective tools to identify various monitoring risks and to combat these risks are immediately required. An automated monitoring system (ASS) can identify health-related risk incidences and prevent them.

Many researchers have utilized deep learning models for the monotonous prediction of unusual monitoring movements for hospitalized elderly patients [1]. Many monitoring systems employ deep learning models, especially in the image recognition paradigm [2,3], and motion analysis [3,4,5]. Machine learning techniques usually have high prediction performance, such as DNN recurrent neural network (RNN) and Catboost [6]. However, the prediction accuracy has room for improvement. Deep learning techniques are performed by using neurons to extract hidden monitoring features. However, techniques such as deep learning [5,6,7,8,9] take a long time to train the system. Thus, a single deep learning technique will not encounter the real-time requirements of the monitoring systems in the new generation.

There are several hybrid monitoring prediction models that unite several deep learning techniques such as support vector deeps (SVM) and C-means [10,11,12,13], SVM based on naive Bayes, LDBoost [11,12], and support vector deep with recurrent C-means clustering [3]. Also, there are mixtures of several deep learning techniques such as Vinaya-kumar and its equivalents [12,13,14]. The authors in [15] utilized RNN and SVM models to extract spatial and temporal features. The authors in [16] presented a recurrent DNN system to detect the semantic features of monitoring settings. Other unions of deep learning techniques and neural techniques. For instance, the authors in [17] presented a deep learning model with a hidden belief technique to extract features and RNN for classification. The authors in [18] utilized a stacked encoder-decoder model for feature selection and then utilized random forests for classification. The authors in [19] presented a deep learning fusion classification model.

**Figure 1:**
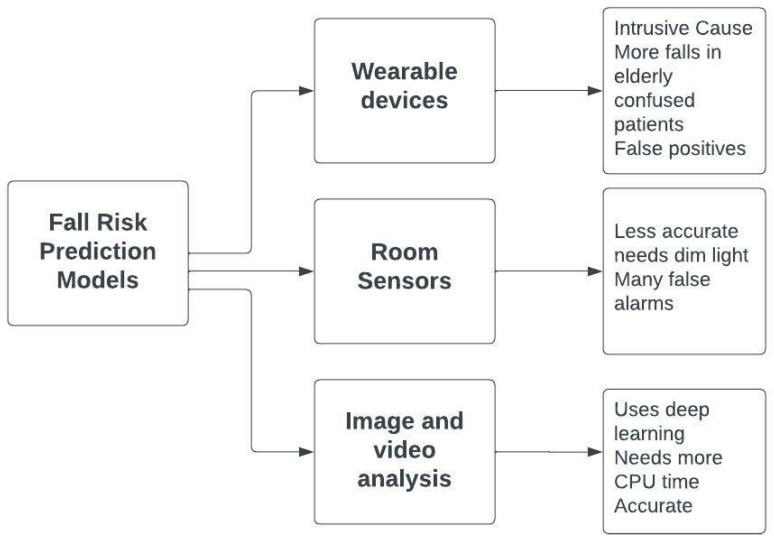
Fall risk prediction models can consist of wearable devices, room sensors or image and video analysis.

Distributed intelligent learning techniques and deep techniques are presented by many researchers [19,20,21,22]. Such systems train high-dimensionality input rapidly and effectively so they can be utilized to train the enormous size of data in the training phase of the two phase prediction monitoring systems. Multi-risk prediction phase of monitoring systems, deep neural techniques can extract hidden features and extract unpreceded health-related risks with high performance. An investigation of monitoring prediction is proposed in [19]. The authors in [19] presented a flow model which performed the first phase by using Snowflake Python as the deep learning framework and the RNN architecture. The complexity is enlarged if the data is altered on several hardware models. Such methods predict only safe and risky incidences without further classifying risky incidences. These hybrid models are doing well on previously defined datasets, but with new health-related risk types, more development is necessary. Guaranteeing the real-time requirements of monitoring prediction without degrading the performance is an imperative problem that has to be deliberated for next-era monitoring systems. Based on previous research, this paper proposes an improvement for an automated health monitoring system such that various risky incidences can be classified in real-time, thus constructing a more reliable system. Different fall risk prediction models are depicted in Figure 1.

Therefore, in this paper, we introduce a monitoring intelligent prediction system. The proposed system incorporates a scalable clustering method using the Catboost binary classification. These techniques are executed on the Snowflake platform (https://www.snowflake.com/en/) accessed on 23 June 2022, for real-time prediction of risk. Snowflake’s Data Cloud is powered by an advanced data platform provided as Software-as-a-Service (SaaS). Snowflake enables data storage, processing, and analytic solutions that are faster, easier to use, and far more flexible than traditional offerings [22] We then employ a deep learning neural network to classify various monitoring alert types (fall, fall with broken bones, and falls that lead to death).

This paper proposes a multi-stage monitoring prediction model using a distributed method that can be utilized for a considerably sized dataset. The proposed model utilizes intelligent techniques to extract hidden features to prevent an overfitting problem. A comparison of the performance of the true positive rate on the SERV-112 and SV-S2017 datasets depicts a higher true positive rate for health-related risk incidences and a higher true positive rate for multi-risk prediction. Both datasets are composed of an image sequence of 120 frames for each incident.

The organization of this paper is depicted as follows. Section 2 outlines the materials and methods of the proposed research. The experimental settings and the classification results are elucidated in Section 3. Conclusions from the proposed research are perceived in Section 4.

## 2. Materials and Methods

**Figure 2:**
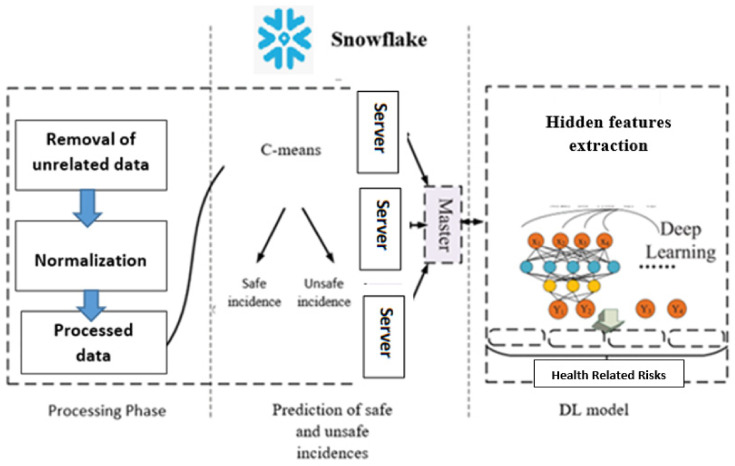
The three phases of the monitoring and prediction framework. The first phase of the monitoring prediction comprises the analysis of the class attributes with data processing and the removal of unrelated data. Furthermore, normalization is performed to obtain the processed data. The second phase predicts safe and unsafe incidences based on the scalable clustering algorithm. In the final phase, the hidden features are extracted using DL techniques.

The proposed monitoring/prediction system comprises three phases. The first phase of the monitoring prediction involves the analysis of the class attributes with data processing and the removal of unrelated data. In addition, normalization is performed to obtain the processed data. Phase 2 predicts safe and unsafe incidences based on the scalable clustering algorithm. In the final phase, the hidden features are extracted using DL techniques. As depicted in Figure 2, Snowflake is the core of the presented system. Before prediction, the conforming libraries of Snowflake have to be imported. Snowflake for the experiment settings of this research. The training dataset in the last phase is deducted from the sampling by SMOTE [20] after eliminating safe incidences in the first phase. The testing subsets of the last phase are deducted from the rest of the results from the testing datasets.

### 2.1. Distributed C-Means Phase (DC)

The main stages of the C-means technique are to choose cluster centroids, compute the displacements between incidence points and the cluster centroids, then allocate each incidence to the correct centroid. Stages will be reiterated to check that the state is attained. The DC technique computes the displacement between the cluster centroid and the incidence by computing the Euclidean displacement, which is defined as the matrices of the squared distances between points [21]. The formula is calculated as follows:

(1) $$dis\left(P1,P2\right)=\sqrt[{\;}]{{\left(P11-P21\right)}^{2}+{\left(P12-P22\right)}^{2}+\cdots \cdots +{\left(P1m-P2m\right)}^{2}}$$

where P1 and P2 represent two incidences with m fields and dis(P1,P2)= represents the Euclidean displacement between points P1=(P11,P12,…,P1m) and P2=(P21,P22,……,P2m). The experiments are performed in Snowflake. The data is portioned into several subsets which relate to various distributed sets (DSets). Each DSet saves multiple subsets. The C-means algorithm groups the subsets in each DSet and computes the concluding results. Snowflake will perform several threads to calculate the subsets in the servers. The preliminary cluster centroid is 9, so the concluding result is 9.

### 2.2. Distributed Catboost Technique

The Catboost technique is a unified deep learning technique that constructs forest trees using node splitting and reselection. The ultimate prediction outcome is nominated from the results of several trees [23]. Following the C-means phase, safe incidences and unsafe incidences will be classified by Catboost, and these unsafe incidences will be further partitioned. Catboost performs training on a group of forests disjointedly, so the learning process can be performed on Snowflake. The forests are used to construe DSets. The essential part is fixing the count of the forests rationally. The count of forests is 500 with the longest one being 30 levels. The clustering forest technique of the Catboost technique is designated in the following equation:

(2) $$R\left(P\right)=argmax_{P}(\overset{n}{\underset{k=0}{\sum }}Prob\;\left(r_{i}\left(P\right)=A\right)$$

where, *P* and *A* denotes a particular prediction incidence and object, where $r_{i}\left(P\right)$ denotes a prediction result, $Prob\;\left(r_{i}\left(P\right)=A\right)$ is the probability computation, and R(P) denotes the prediction of the Catboost technique.

### 2.3. Deep Learning Techniques for Multi-Risk Prediction

**Figure 3:**
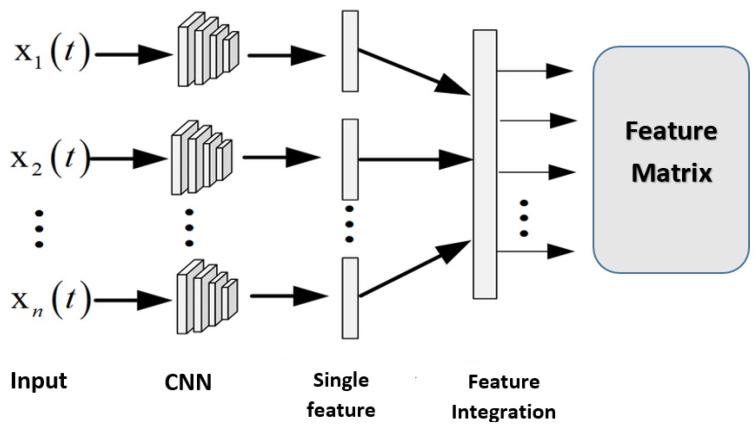
Architecture of the proposed DNN (inputs are fed in parallel to the convolutional neural networks where features are extracted and then integrated through the feature integration phase to produce the feature matrix).

In the final phase, the deep learning technique (DNN) is utilized to accomplish multi-risk classification. DNN usually comprises an input, deep learning, pooling, and fully connected layers (FC). They are feedforward neurons that employ the convolution operation. The convolution operation selects hidden features, and the kernels achieve convolution computation with the initial data to select those key features. DNN is partitioned into several parallel CNNs, with each CNN extracting a single feature from a subset of the input. Feature integration is performed to output the required feature matrix as depicted in Figure 3. In this article, health-related risk incidences can be classified into safe incidences and unsafe incidences.

The deep learning technique (DL) is utilized to accomplish multi-risk irregularity classification. DL usually comprises an input, deep learning, pooling, and fully connected layers (FC). They are feedforward neurons that employ the convolution operation. The convolution operation selects hidden features, and the kernels achieve convolution computation with the initial data to select those key features. DL is partitioned into DL-1D, DL-2D, and DL-3D using the input. Several dimension layers have various scenarios. DL-1D is utilized in serialization systems. In this article, health-related risk incidences can be classified into safe incidences and unsafe incidences. Thus, DL-1D can be utilized as the monitoring prediction system. The forward network of the DL convolution can be computed as depicted below:

(3) $$i^{l}=ReLU\;\left(i^{l+1}\times \omega^{l}+O^{l}\right)$$

(4) $$ReLU\;\left(i\right)=\{\begin{array}{c} i,\;i>0\\ 0,\;i<0\; \end{array}$$

where ReLU is the rectified linear operation unit. The computation is depicted in Equation (4), where $i^{l+1}$ and $i^{l}$ denote the input and the output of the neural operation. $\omega^{l}$ denotes the $l^{th}$ layer weight vector, and $O^{l}$ denotes the offset of the $l^{th}$ layer.

DL is intended to tackle the gradient fading problem of the RNNs. The proposed DL model has a direct link between earlier and late stages of the monitoring prediction incidence; this research uses the time series system. A standard deep learning unit is composed of an input cell 9 that has an image In(x,y), an output cell (has an output image Out(x,y), and an ignore cell (with an image Ig(x,y)). As depicted below:

(5) $$In\left(x,y\right)=\frac{{pix}^{\prime }\left(j,k\right)-pix_{min}}{pix_{avg}-pix_{min}}$$

(6) $${pix}^{\prime }\left(x,y\right)=\frac{1}{r*r\;}\;\overset{x+r/2}{\underset{X=x-r/2}{\sum }}\sum_{Y=y=r/2}^{y+r/2}pix\left(x,y\right))$$

(7) $$In\left(x,y\right)=\frac{\xi \left(x,y\right)-\xi min}{\xi max-\xi min}$$

(8) $$\xi \left(x,y\right)=abs(pix\left(x,y\right)-pix^{\prime }\left(x,y\right),\;Ig\left(x,y\right)=1-\psi \left(x,y\right)$$

Since ${pix}^{\prime }\left(x,y\right)$ is a local optimum and ξ(x,y) is the absolute difference between pix(x,y) and ${pix}^{\prime }\left(x,y\right)$. pix(x,y) represents the pixel value at points *x* and *y*.

The entropy of the energy is computed for the positive set, ignore set, and negative set as depicted below. Where prob depicts the probability for an incidence *Z*.

(9) $$Entropy\;\left(Positive\right)=-\overset{\max\left\{Positive\right\}}{\underset{Z=\min\left\{Positive\right\}}{\sum }}prob\left(Z\right)\ln\left(prob\left(Z\right)\right)$$

(10) $$Entropy\;\left(Ignore\right)=-\overset{\max\left\{Ignore\right\}}{\underset{Z=\min\left\{Ignore\right\}}{\sum }}prob\left(Z\right)\ln\left(prob\left(Z\right)\right)$$

(11) $$Entropy\;\left(Negative\right)=-\overset{\max\left\{Negative\right\}}{\underset{Z=\min\left\{Negative\right\}}{\sum }}prob\left(Z\right)\ln\left(prob\left(Z\right)\right)$$

(12) Total (Entropy)=Entropy (Positive)+Entropy (Ignore)+Entropy (Negative)

### 2.4. Dataset Description

**Figure 4:**
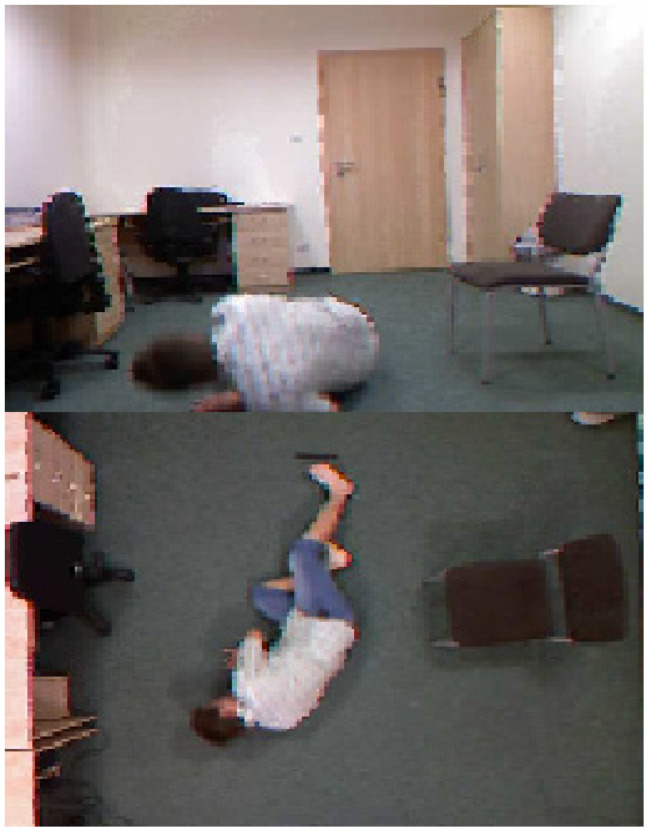
Example of fall images in the datasets SV-S2017.

The benchmark datasets [24,25] are composed of files of extracted features from labelled video sequences captured by cameras in the patients’ hospital rooms. These videos undergo splitting into frames which are fed to the deep learning model. The key point is the extracted feature map and the frame labels. These maps are utilized as training data and fed to the fall monitoring deep network. The output model then classifies the fall of the testing frames for new incidences. The SERV-112 [24] and the SV-S2017 [25] datasets are utilized to validate the efficiency of the system presented in this research. The SERV-112 dataset is preprocessed, prearranged and labelled. SV-S2017 was produced in 2019 [22,23,24,25,26] by the Hospitalized Monitoring and European health monitoring. The SERV-112 dataset has labeled image sequences of 30 different features. The extracted features in the training phase of the monitoring prediction system are the entropy functions and the probability functions of different classes. The SV-S2017 dataset utilized in this research has continuous-valued features such as optical flow features. Examples of fall images from the dataset SV-S2017 are shown in Figure 4.

Description of the datasets according to labelled fall cases (safe, fall, fall with broken bones, fall that leads to death) are depicted in Table 1 and Table 2. The distribution of both data sets for the training, validation, and testing datasets is depicted in Table 3.

#### 2.4.1. Feature Extraction

Health issue group, risk, and red flags define the categorical reported attributes in SERV-112. The attributes are transformed into values. For instance, the feature of the health issue type includes severely paralyzed, moderately paralyzed, and non-paralyzed, which is transformed into ordinal values from one to three. In this situation, the health issue type feature is transformed to ordinal values [1, 60].

#### 2.4.2. Data Normalization

Data normalization is the computation to convert the multi-dimensional feature into a one-dimensional value. Preprocessing confines the values to definite ranges to reduce the effects done by the difference in variance values. In this article, all representative values are normalized to [0, 1], where $f_{m,n}$ denotes the $n_{th}$ feature of the $m_{th}$ incidence.

(13) $$f_{m,n}=\frac{f_{m,n}-Min}{Max-Min}$$

where Max is the greatest value of the $n_{th}$ feature, and Min is the smallest value of the $n_{th}$ feature.

#### 2.4.3. Label Binarization

The incidence labels of the SERV-112 and SV-S2017 are numeric values, and binarization labeling of is utilized to define categories into vectors of bits. In the SERV-112 dataset, we have eight categories representing the severity of immobility. In the SV-S2017 dataset, we have the same eight categories, in addition to two categories of health-related risks, namely previous injuries and previous falls. This research performs label binarization for the immobility features and the health-related risks categories.

### 2.5. Bi-Classification Using Distributed Deep Learning

**Figure 5:**
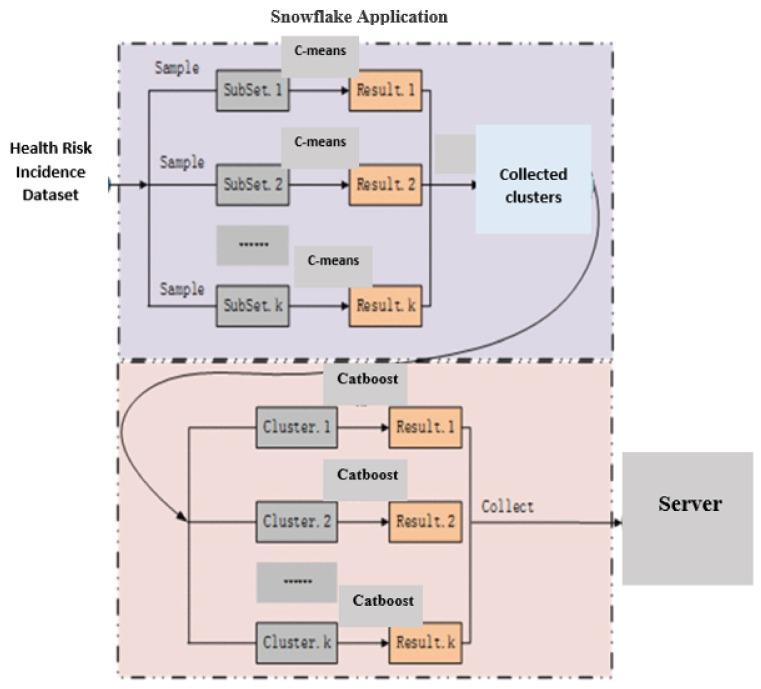
The distributed binary monitoring prediction system. Health risk incidences are inputted to the distributed training system and the results are computed and gathered in the first phase. The gathered clusters are fed to the Catboost classifiers in the second phase and collected and sent to the server.

The second phase is for rapid prediction of health-related risk incidences while guaranteeing the performance of health-related risk incidences. If a high number of health-related risk incidences are misclassified, the monitoring system’s false negative count will rise, and the monitoring systems will not be operative. Thus, the objective of this phase is to isolate the health-related risks promptly. The enhancement of the monitoring system’s performance is governed by the legitimacy of the input. The data composed by the monitoring systems are diverse collected from patients’ behavior and aptitude. This multiplicity that we employ can produce the operative processing of monitoring incidences. These data of multi-dimensional monitoring systems need to predict the risky incidence before classifying health-related risk classes. The health-related risk is clarified by removing safe incidence, which decreases the effort and time complexity of the in-depth analysis of health-related risks in the final phase. The prediction at this phase is depicted in Figure 5.

The binary monitoring prediction system integrates the DC technique and the Catboost technique using Snowflake. The DC technique is utilized to group monitoring prediction incidences, and Catboost is utilized to categorize the clusters. The input is separated into several subsets according to the structure of the Snowflake system and the data size. All different data subsets subsist as DSet in Snowflake. Each transmutes and action function in the technique will be incidences and will be applied to all DSets. Thus, the incidence sets in Figure 5 are partitioned into several subsets. In the procedure, the C-means technique is utilized to cluster the input to form clusters. At that juncture, the DSet aggregation operation is utilized to combine the clusters into the results sets. For $Result_{1}$, $Result_{2}$, and $Result_{3}$, until the last cluster, several schemes are employed using different points. Clusters with less than thirty points are either classified as unsafe or the Catboost technique is utilized to predict the results.

### 2.6. Multi-Risk Health Related Risk Incidences Classification Using Deep Learning Technique

In the last phase, the incidences classified as safe by the Catboost technique in the previous phase will leave the system directly. Many health-related risk incidences are positively separated, and the incidences classified as unsafe will enter the last phase. Using these schemes, unsafe incidences and safe incidences can be extracted. The model platform of the DC and the Catboost techniques are depicted in Figure 5. The point limit in Figure 5 is 30 as depicted.

#### Monitoring Prediction Deep Learning System Framework with Long Short-Term Memory (LS)

**Figure 6:**
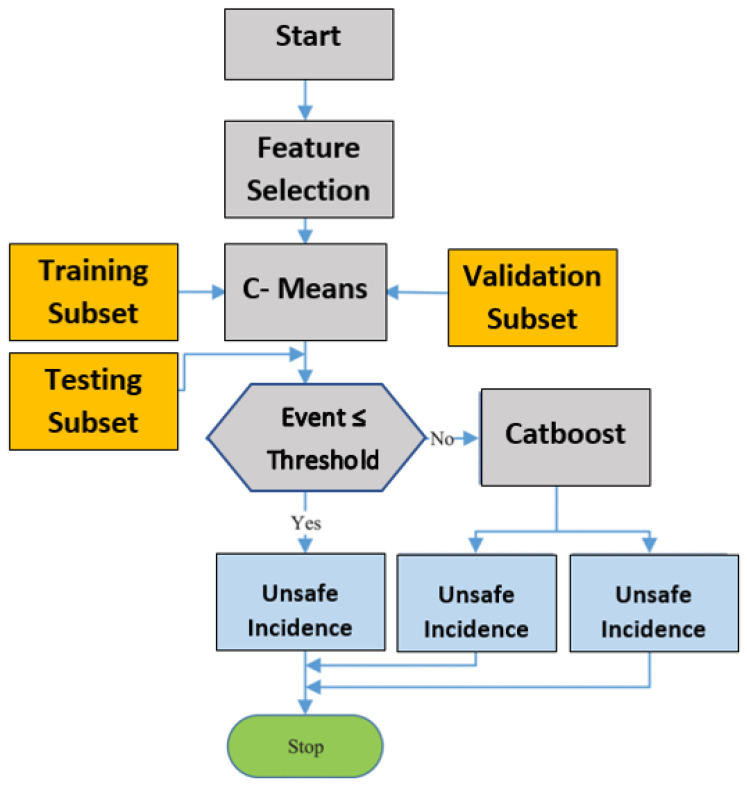
The model platform of the C-means and the Catboost techniques. The model depicts the training and testing set to produce the different safe and fall classes.

In this phase, three architectures are used, namely: “DNN”, “LS”, and “DNN + LS”, and are utilized to validate the accuracy of the system. Each system structure comprises from one to three hidden layers. ReLU classification is used, and the final phase is fully connected (FC). The classification is performed using Softmax. These structures are utilized to discover the most appropriate multiple risk health-related incidence prediction method. All health-related risk incidences are classified using Softmax. The whole configuration of this phase is depicted. In the last preprocessing stage, SERV-112 has 120 attributes and SV-S2017 has 80 attributes. The last layer has six dense layers for the SERV-112 dataset and eight in the SV-S2017 dataset, as depicted in Figure 6.

## 3. Results and Discussion

The deep learning model is depicted in Table 4. We generated a Snowflake framework and ran Snowflake processes; this is also an essential experimental setting for the final phase. The nodes are configured with a TMS 34010, Intel, New York, NY, USA, graphic processor (https://www.ti.com/lit/pdf/spvu015 accessed on 23 June 2022), which utilizes Tensor as the prime platform of Keras. This permits us to decrease the training time of the DNN system. After the binary prediction by the DNN model on the Snowflake framework, the safe and unsafe incidences are directly computed by the distributed model to the master side. In the final phase, the monitoring prediction data saved on the master side can be utilized for prediction straight away as depicted in Table 4.

### 3.1. Evaluation

Evaluation methods for prediction results of unsafe incidences are used. Precision and true positive rate and true negative rate are mostly measured. The ratio of incidences in the test subset diverges, and the classification outcomes may be inclined to incidences with higher occurrence data. Thus, the precision is not enough. The classification outcomes produced by the monitoring system can be partitioned into several groups.

The accuracy is the percentage of correctly classified incidences to the total incidences count. Accuracy is employed for all safe incidences and unsafe incidences in the test dataset. The true positive rate is the percentage of incidences defined as health-related risks, which are properly classified as health-related risks in the datasets for all items defined as health-related risks as depicted in the equations below.

(14) $$\mathrm{Accuracy}\;=\frac{TP+TN}{TP+FP+FN+TN}$$

(15) $$\mathrm{True}\;\mathrm{positive}\;\mathrm{rate}=\frac{TP}{TP+FN}$$

(16) $$\mathrm{True}\;\mathrm{negative}\;\mathrm{rate}=\frac{TN}{FP+TN}$$

where, true positive (*TP*) is defined as the correctly classified health-related risk incidences, and *TN* denotes the properly classifies safe incidences. *FP* depicts the mistakenly classified positive incidences and a false negative is defined as the incorrectly classified negative incidences.

### 3.2. Binary Prediction Experiments

The prediction by the deep DNN prediction system, the results are depicted in Table 5 and Table 6. The prediction rate in SERV-112 is 91.80%, and the prediction results in the SV-S2017 are 99.67%, specifying health-related risk incidences can be predicted accurately. The false positive rate of health-related risk incidences is very little. This indicates that the accuracy of the prediction system is high. Prediction accuracy and the positive ratio for the SERV-112 set can encounter further prediction of health-related risk incidences in the final phase.

### 3.3. The Experimental Results of the Multi-Risk Health Related Incidences Prediction

The multi-risk health-related incidences prediction employs several deep learning frameworks: DNN, long short-term memory, and DNN + long short-term memory. The factors of the training system affect the prediction experimental ratios. After several epochs, the deep layers of the DNN system are made equal to 2056, the FC size is made equal to 6, and the pooling layers are made equal to 4. The factors for the long short-term memory system are set to 520. The parameter of the DNN + long short-term memory system is computed using the previous systems. The preliminary learning rate is equal to 0.02.

The precision rate of the loss rate of the training systems of eight convolutions is depicted. The three deep learning systems can accomplish high accuracy and low loss rates. It is observed that the count of epochs needed to predict only health-related risk incidences is 100 epochs, which is considerably low compared to the 500 epochs needed by the model in [27] to predict safe incidences and the five types of health-related risk incidences. The early splitting of safe incidences avoids pointless training time for the multi-risk unsafe prediction phase based on the training deep learning CNN techniques [28,29,30]. Therefore, the learning curve can be considerably abridged [31].

In [32,33] the study assessed Tresca stress in CoCrMo-onCoCrMo hip implants using patient body mass index. The authors presented a 2D computational model to attain this objective.

**Figure 7:**
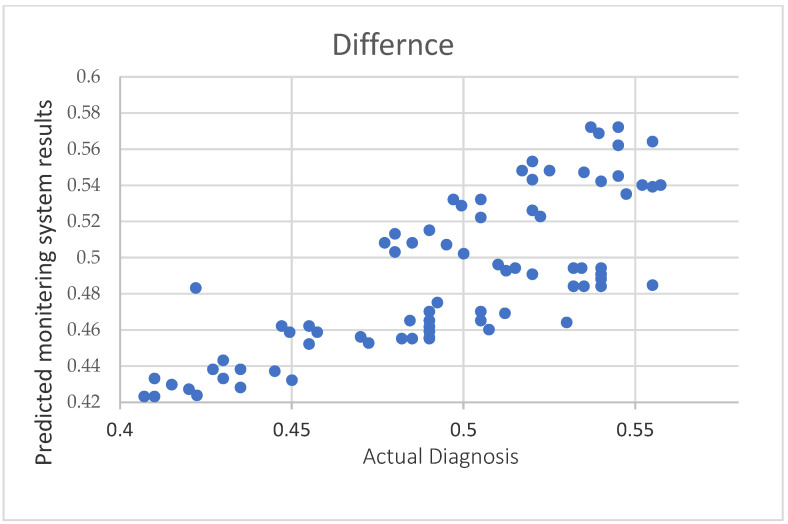
The correlation of the real ratio of health risk incidence in cases from a medical diagnosis versus the prediction of risk incidence from the proposed monitoring system.

Results depicted in Figure 7 show the correlation between the real ratio of risk incidence in elderly patients from a physician’s judgment from patients’ charts, and the ratio of the risk incidence detected from the proposed system.

**Figure 8:**
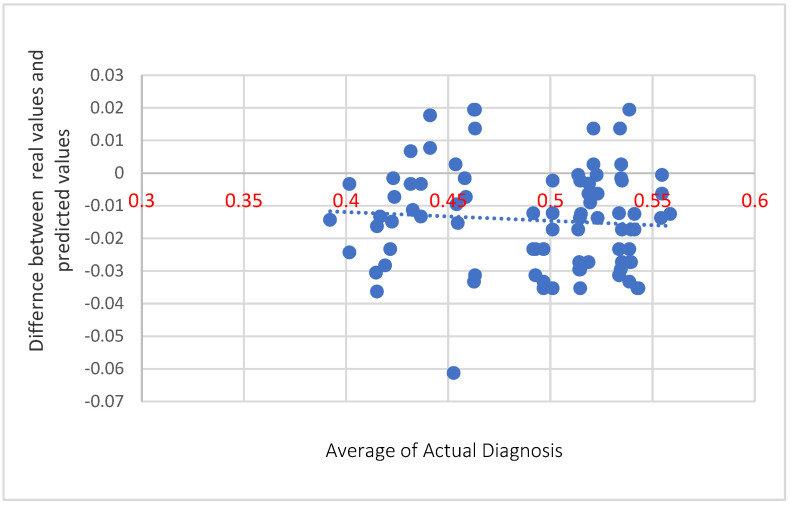
The Bland-Altman Plot that denotes the difference of the predicted values versus the average of actual diagnoses.

The Bland-Altman plot of the real ratio of the risk incidence in elderly patients from a medical personal prognosis in comparison to the calculated ratio of risk incidence of the proposed system is depicted in Figure 8.

The true positive rates of health-related risk incidences used for the three used models are depicted in Table 5 and Table 6. In the SERV-112 dataset, the monitoring prediction method predicted by the DNN is comparatively balanced. The True positive rate in the DNN system with four hidden layers is above 80%, and the health-related risk incidences have a moderate recall rate. Compared with the system presented, long short-term memory is considerably enhanced in the proposed research, with a true positive rate as high as 92.8%. The other groups of health-related risk incidences have a reasonably balanced true positive rate (namely; fall, fall with broken bones, and fall that leads to death). Table 7 shows that other common health-related risk types have the highest true positive rate in the presented methods in the SV-S2017 dataset, except for falls that lead to death risk. In precise, the True positive rate of Fall, Fall with broken bones accomplish up to 98.7%. This means that fall and fall with broken bones can be classified with high precision, as depicted in Table 7 and Table 8.

**Figure 9:**
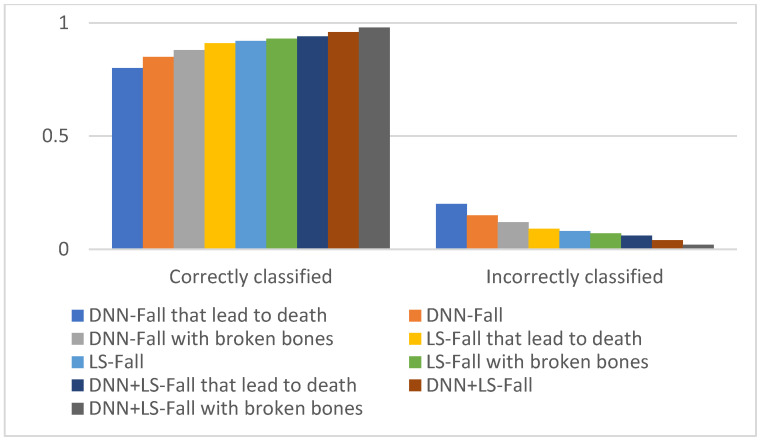
Correctly classified versus incorrectly-classified instances.

The computational load is challenging due to the fact that these systems were realized on various platforms. Thus, the computational load can be depicted by simulation models. The final phase of the system is grounded on the statistic that testing datasets are partitioned into unsafe related risk incidences and safe incidences from the second phase. The incidences predicted as unsafe-related risks from the second phase were gathered on the server. The DNN prediction system of the final phase is predicted from the server, and testing will be converted frequently on various platforms. Snowflakes’ distributed hardware finalizes the data of the training input in the final phase and will save training time in this phase. The prediction system utilizes Azure Machine, which utilizes the speedup technique for faster training, so the computational load of the final phase can be enhanced. Figure 9 depicts the correctly classified versus incorrectly-classified instances. Table 9 and Table 10 depict the comparison of the prediction time in seconds.

### 3.4. Comparison and Discussion

Comparison between the proposed model (DNN + LS) model and other fall prediction models is displayed in Table 11.

#### Computational CPU Time

In deep learning neural networks, training time is one of the metrics for defining the model performance. In addition, classification CPU time is very crucial, especially for real time applications. The fall monitoring system is one of the real time applications where classification has to be done in real time from video captured by the camera. In the following table we display the CPU time comparisons for both training time and classification time.

As we can see from Table 12, the proposed model maintains an average time that is lower than the other models in training time. This is due to two factors: The first factor is the Snowflake’s distributed hardware where the system is designed and implemented to be distributed system. The second factor is the application of the C-Means from the beginning to cluster the fall incidence from the No-Fall incidences before the deep learning training phase. The same two factors contribute significantly to less classification time for alarms to notify health personnel about a future fall incident, as depicted in Table 13.

## 4. Conclusions

This article presented a cascading monitoring prediction technique based on distributed C-means, Catboost, and deep learning. We presented a methodology to solve monitoring systems problems that are computationally time-consuming and that have low prediction accuracy. The distributed methodology is utilized to accomplish the time-efficient processing of the monitoring prediction dataset. The unsafe related risk incidences and safe incidences were alienated by incorporating the distributed C-means and the Catboost technique. The isolated unsafe related risk incidences are fed to deep learning systems that extract hidden features of multi-risk incidences. The final phase performs the prediction of various related risk incidences promptly. The prediction system presented in this paper is evaluated using the SERV-112 and SV-S2017 datasets. The experimental results depict that the presented method can efficiently recognize the prediction of health-related risk incidences. The performance of safe incidences and the other three types of unsafe related risk incidences is 87.24% in the SERV-112 dataset, while the accuracy of unsafe incidences is 98.9% in the SV-S2017 dataset. We also compared the proposed model (DNN + LS) with the separate deep learning monitoring prediction systems (DNN and LS)), and the system presented in this paper achieved a higher true positive rate for most risk incidences irrespective of the incidence number of these related risk incidences, which determined that more elderly related risks could be precisely predicted. The proposed model maintains average time that is lower than the other models in training time. This is due to two factors. The first factor is the Snowflakes’ distributed hardware where the proposed system is designed and implemented to the distributed system. The second factor is the application of the C-Means from the beginning to cluster the fall incidence from the No-Fall incidences before the deep learning training phase. The same two factors contribute significantly to less classification time for alarms to notify health personnel about a future fall incident.

The limitation of this study is the lack of posture information in the learning stage, as it might help in the classification process, but it might take require more classification CPU time.

## Figures and Tables

**Table 1:** Description of the datasets according to safe, fall, falls with broken bones, fall that leads to death cases in the SERV-112 dataset.

Label	Number of Images	Percentage %
Safe	2010	21.60
Fall	2565	27.57
Falls with broken bones	2170	23.32
Fall that leads to death (no movement for the whole sequence)	2560	27.51
Total	9305	

**Table 2:** Description of the datasets according to safe, fall, falls with broken bones, falls that lead to death cases in the SV-S2017 dataset.

Label	Number of Images	Percentage %
Safe	2300	23.69
Fall	2611	26.90
Falls with broken bones	2337	24.07
Fall that lead to death	2460	25.34
Total	9708	

**Table 3:** The distribution of both data sets for the training, validation, and testing datasets where 70% of both datasets is used for training, 15% for validation and 15% for testing.

	SERV-112 Dataset	SV-S2017 Dataset	Percentage
Training	6515	6796	70%
Validation	1395	1456	15%
Testing	1395	1456	15%
Total	9305	9708	

**Table 4:** Experiments Configuration of the neural network with the structure of the CNN layers.

Layer	Layer Type	Structure
1	Input Layer	1024 × 1024 × 3
2	Convolution	512 × 32 × 1
3	Pooling	Max pooling
4	Convolution	256 (8 × 8 × 3)
5	Second Pooling	Average
6	Convolution	64 (3 × 3 × 3)
7	Fully Connected (FC)	2020 neurons
8	Classifier	Softmax

**Table 5:** The confusion matrix denotes the accuracy of the model using the SV-S2017 set.

		Predicted Cases	
		Positive	Negative
Actual cases	Positive	1010	12
	Negative	8	772

**Table 6:** The confusion matrix denotes the accuracy of the model using the SERV-112 set.

		Predicted Cases	
		Positive	Negative
Actual cases	Positive	900	82
	Negative	30	920

**Table 7:** Comparison of performance results with different numbers of clusters from the C-Means (N) for the SV-S2017 set.

	DNN			LS			DNN + LS		
N	Precision	TP	TN	Precision	TP	TN	Precision	TP	TN
3	0.82	0.84	0.80	0.91	0.90	0.90	0.93	0.93	0.92
4	0.82	0.89	0.82	0.91	0.92	0.92	0.925	0.93	0.91
5	0.84	0.84	0.84	0.95	0.92	0.90	0.925	0.935	0.93
6	0.86	0.86	0.86	0.92	0.92	0.92	0.93	0.925	0.945
2	0.83	0.83	0.83	0.92	0.91	0.95	0.93	0.933	0.936
8	0.89	0.80	0.89	0.91	0.92	0.91	0.93	0.93	0.937

**Table 8:** Comparison of performance results with different numbers of clusters from the C-Means (N) for the SV-S2017 set.

	DNN			LS			DNN + LS		
N	Precision	TP	TN	Precision	TP	TN	Precision	TP	TN
3	0.92	0.94	0.90	0.93	0.90	0.96	0.98	0.99	0.98
4	0.92	0.99	0.92	0.91	0.92	0.93	0.97	0.98	0.99
5	0.94	0.94	0.94	0.95	0.94	0.96	0.97	0.96	0.99
6	0.96	0.96	0.96	0.92	0.94	0.92	0.98	0.97	0.98
2	0.93	0.93	0.93	0.94	0.96	0.95	0.98	0.99	0.98
8	0.99	0.90	0.99	0.91	0.92	0.91	0.98	0.98	0.98

**Table 9:** Statistics for using DNN model and C-Means with a long short memory for multi-risk prediction.

	Fall That Leads to Death	Fall	Fall with Broken Bones
TP	0.841	0.912	0.96
FP	0.159	0.088	0.04
Inter-Qualitative Reliability	0.187	0.201	0.314
Absolute Square Error	0.841	0.521	0.311

**Table 10:** Comparison of the prediction time in seconds.

	Method	Execution Time (s)
Binary Classification	DNN	14.42 ± 0.115
	LS	19.25 ± 0.429
	DNN + LS	4.42 ± 0.329
Multi Classification	DNN	9.32 ± 0.223
	LS	8.29 ± 0.929
	DNN + LS	7.42 ± 1.029

**Table 11:** Precision comparison averaged on both datasets.

Method	Classification	Average Accuracy %	Average Sensitivity %	Average Specificity %
The proposed DNN model and C-Means with a Long short memory for multi-risk prediction	Multi classes	97.6%	98.1%	97.9%
A Lightweight Subgraph-Based Deep Learning Approach for Fall Recognition [2]	Binary classification (Fall, No-Fall)	97%	98%	96%
Human Fall Detection Based on Pose Estimation [30]	Three classes (Fall, No-Fall, Unknown)	93.4%	94.6%	92.1%

**Table 12:** Training CPU time of training of all methods.

Method	(Average Minutes)	Standard Deviation
The proposed DNN model and C-Means with a long short memory for multi-risk prediction	152	±9.9
A lightweight subgraph-based deep learning approach for fall recognition [2]	560	±9.6
Human fall detection based on pose estimation [30]	509	±10.9

**Table 13:** Classification CPU time of training of all methods.

Method	(Average Seconds)	Standard Deviation
The proposed DNN model and C-means with a long short memory for multi-risk prediction	0.67	±0.12
A lightweight subgraph-based deep learning approach for fall recognition [2]	5.6	±0.6
Human fall detection based on pose estimation [30]	9.5	±0.9
